# Designing for self-regulation: development and preliminary user evaluation of a student-facing learning analytics app in blended higher education

**DOI:** 10.3389/fpsyg.2026.1914516

**Published:** 2026-09-14

**Authors:** Karla Lobos, Rubia Cobo-Rendón, César Mora, Nelson Arias, Carolyn Fernández, Camilo Abrigo

**Affiliations:** 1 Dirección de Docencia, Universidad de Concepción, Concepción, Chile; 2 Laboratorio de Aprendizaje Multinivel en Educación Superior (AMES), Instituto de Bienestar Socioemocional (IBEM), Facultad de Psicología, Universidad del Desarrollo, Concepción, Chile; 3 Faculty of Engineering, Universidad de Concepción, Concepción, Chile

**Keywords:** blended learning, higher education, learning analytics, self-regulated learning, technology acceptance

## Abstract

**Background:**

Blended learning environments place substantial demands on students’ capacity to plan, monitor, and evaluate their own academic work, yet most technology-mediated interventions in higher education prioritize instructor-led monitoring over student-centered autonomy.

**Objectives:**

This study aimed to characterize the challenges and self-regulated learning strategies of university students in blended learning contexts, design and validate a student-oriented learning analytics application natively integrated into a learning management system and explore the perceived experience and technological acceptability.

**Design:**

An exploratory sequential mixed-methods design was employed. Participants: The qualitative phase included semi-structured interviews with 14 faculty members and 19 focus groups with 140 students (mean age = 21.53, SD = 2.87) from three Chilean universities, complemented by expert review conducted by seven specialists in educational technology; the quantitative phase involved two successive, non-equivalent pilot implementations with 5 faculty and 267 students enrolled in first-year high-risk courses. A 20-item Likert-type scale incorporating elements on self-regulation of learning and the Technology Acceptance Model was administered after each implementation. Qualitative data were analyzed through qualitative content analysis with inductive orientation; quantitative data were examined descriptively, with no inferential comparisons between pilots given differences in sample size and context.

**Results:**

Five overarching challenge domains were identified alongside four student strategy clusters. The final application, comprising seven interfaces aligned with Zimmerman’s three-phase model of self-regulated learning, received descriptively higher perceived impact ratings in the final pilot (forethought: *M* = 4.0–4.8; performance: *M* = 3.7–4.4; self-reflection: *M* = 3.7–4.4) than the preliminary version (all dimensions *M* = 2.2–3.0), with higher perceived usefulness (*M* = 4.1) and perceived ease of use (*M* = 3.8) than in the preliminary version.

**Conclusion:**

These exploratory findings suggest that student-facing learning analytics, when embedded within institutional virtual classrooms, may be associated with higher perceived support for self-regulatory processes across all three phases of the model, positioning students as active agents in interpreting their own learning data rather than passive subjects of instructor-facing monitoring systems. These findings should be considered preliminary and hypothesis-generating rather than confirmatory.

## 1 Introduction

Online learning is defined as a process that separates students, faculty members, and course materials from the traditional educational environment, shifting learning activities entirely or partially to a digital space supported by educational technologies (Hodges et al., 2020; Nouraey et al., 2023). This phenomenon, initially a supplement to face-to-face instruction, became firmly established following the COVID-19 pandemic, marking a turning point in how higher education institutions conceive of teaching and learning. In the post-pandemic scenario, the global trend has been to leverage the lessons learned during the health crisis by expanding the use of learning management systems (LMS) and their virtual classrooms as structural components of instruction (Lederman, 2020; Pardo Kuklinski and Cobo, 2020). Within this landscape, blended learning (BL) has gained prominence, not as a complete migration to virtual settings, but as a flexible instructional model that combines face-to-face and digital environments to optimize interaction and overcome traditional barriers of space and access (Hrastinski, 2019; Singh and Thurman, 2019). Its potential lies in fostering meaningful interaction and a more active student orientation toward learning (Freeman et al., 2014); yet it simultaneously places specific and substantial demands on students regarding personal organization, autonomy, and self-directed study management.

Meeting these demands requires strong self-regulated learning (SRL) competencies. Zimmerman's (2002) influential model describes SRL as three interrelated phases, forethought, performance, and self-reflection, through which students set goals, organize resources, monitor their progress, and critically evaluate their outcomes. Research consistently documents, however, that university students in blended and remote contexts struggle to enact these phases without structured support. Internationally, this pattern is well established across diverse contexts. In the Middle East, a study with 144 university students found that those with greater SRL skills achieved markedly better academic performance during pandemic-related lockdowns (Ibrahem Qetesh et al., 2020). In the United States, a study with 565 students revealed that the virtual environment led many to adopt strategies focused on passing with minimal effort, prioritizing efficiency over deep learning (Zhang et al., 2021). On a global scale, a survey involving over 30,000 students from 62 countries identified widespread difficulties related to time management and academic organization as primary factors negatively affecting performance during emergency remote education (Aristovnik et al., 2020). In Chile, a survey of 8,265 university students confirmed these difficulties while also yielding an encouraging result: when comparing initial expectations with actual experience of remote education, students reported an improved perception of the quality and benefits of online learning (Lobos et al., 2021a). A parallel finding from Israel, in a study with 1,217 university students, concluded that adaptability and willingness to face the challenges of remote education translated into a more favorable attitude toward online learning, suggesting that developing SRL and adaptation skills can facilitate the transition to sustainable BL models in higher education (Besser et al., 2022; Trautner et al., 2025). Collectively, these findings establish that SRL is not merely a desirable competency in blended higher education but a structural prerequisite for academic success and that its absence produces measurable, cross-nationally consistent harm to student engagement and performance.

Technological interventions designed to address these challenges remain limited. A review of SRL-focused programs indexed in Web of Science and Scopus between 2010 and 2023 identified over 100 documents on SRL interventions in educational contexts, yet only a small subset targeted university students, and the majority were designed around instructor-led monitoring rather than student-driven autonomy (Manso-Vazquez et al., 2018). Two notable exceptions illustrate both the progress and the persisting gaps. NoteMyProgress (Perez-Alvarez et al., 2018), integrated into Moodle, allows students to visualize academic progress relative to instructor-planned course structures; while it reports improvements in SRL strategy use, its heavy reliance on instructor pre-planning restricts student autonomy and limits the degree to which learners can engage with their own data independently. 4Planning (Lobos et al., 2021b; Jaramillo et al., 2022) addresses goal setting, study planning, and self-assessment through face-to-face structured sessions based on Zimmerman’s model, incorporating gamification and motivational resources; however, it lacks real-time integration with learning analytics that would enable students to monitor their own behavioral data as it is generated. These limitations reveal a critical design gap: to our knowledge, few existing tools are conceived from the university student’s perspective that integrate automated, student-facing feedback and promote autonomous exercise of all three phases of self-regulation within the LMS environment itself.

Learning analytics (LA) offers a promising avenue to close this gap. By leveraging behavioral data generated in the virtual classroom, connection time, resource access frequency, activity completion, navigation sequences, LA can provide students with actionable, personalized insights that support metacognitive decision-making (Maldonado-Mahauad et al., 2018; Li et al., 2023). These indicators can be used not only to observe behavioral patterns but also to actively foster the development of SRL strategies through digital tools that present information in a clear, accessible, and actionable way for students to make personal decisions about their learning. To date, however, most LA implementations in higher education have prioritized instructor dashboards and automated alerts rather than direct student access to their own data (Lu et al., 2017; Sonderlund et al., 2018). This orientation, while valuable for institutional monitoring, tends to shift the locus of control away from the learner, reinforcing external management rather than self-regulation. In contrast, when students interact with systems that allow them to monitor their own learning data and adjust their strategies in real time, improvements are observed in both engagement and academic performance (Hilliger et al., 2020a, 2020b). In this sense, LA acts not only as an informational resource but as a metacognitive scaffold that actively supports planning, monitoring, and evaluation under the student’s own control (Lan et al., 2019).

For LA to be truly transformative, it must move toward a student-centered model in which data generated within the LMS are returned to learners in the form of meaningful visualizations and automated feedback (Li et al., 2023). When students engage with their own analytics, the tool shifts from a surveillance mechanism to a form of scaffolding that supports Zimmerman’s cycle: it enables planning based on historical data, allows monitoring during task execution, and provides an objective basis for final self-reflection (Hilliger et al., 2020a). This study responds to the identified gap by reporting the design and evaluation of Regula2, an SRL application natively integrated into an LMS virtual classroom, developed through an iterative, student-facing co-design process. Unlike prior tools, Regula2 provides student-facing analytics across all three phases of Zimmerman’s model, combining behavioral visualizations, goal-setting features, and structured reflection prompts. The study pursued three aims: (a) to characterize the SRL challenges and strategies of students in blended higher education; (b) to design and validate an LMS-integrated SRL application through iterative expert and user consultation; and (c) to evaluate its perceived impact and technological acceptability in authentic classroom implementations. In doing so, this work contributes to the emerging literature on learner-centered LA by exploring how student-facing analytics, embedded in the institutional LMS, may support student agency across all phases of self-regulation, positioning students as active agents in interpreting their own learning data rather than passive subjects of instructor-facing monitoring systems.

## 2 Method

### 2.1 Design

This study employed an exploratory sequential mixed-methods design (Creswell and Plano Clark, 2023). The qualitative phase comprised faculty interviews and student focus groups to characterize SRL challenges and strategies and to inform the functional requirements of the application, followed by expert review of the preliminary prototype. The subsequent quantitative phase involved two successive pilot implementations to evaluate the app’s perceived impact and technological acceptability in authentic LMS-based classrooms. This sequencing was theoretically motivated: qualitative findings directly shaped the functional architecture of Regula2, expert feedback informed functional refinements, and the pilot implementations provided quantitative evidence regarding how students perceived the resulting application. The design thus reflects an iterative, evidence-driven development model consistent with participatory design principles in educational technology (Vladova et al., 2021; Ifenthaler and Yau, 2020).

Mixed-methods integration occurred through sequential connecting and building across stages. Findings from faculty interviews and student focus groups were translated into functional design requirements for Regula2. These requirements informed the preliminary application architecture, which was subsequently examined through expert review. Expert ratings and qualitative comments were used to identify functionality-specific design gaps and guide revisions before Pilot 1. Pilot 1 did not lead to the addition or removal of core functionalities; instead, its results informed usability-oriented refinements to the user interface, including visual hierarchy, screen organization, element positioning, color use, and navigation, while preserving the same functional architecture. Pilot 2 therefore evaluated the same core functionalities through a more intuitive and user-friendly interface. Across stages, integration was examined by linking qualitative requirements, design decisions, expert feedback, and pilot ratings rather than treating each phase as an independent component.

### 2.2 Participants

The research included three groups of participants in the qualitative phase (faculty, students, and experts) and two groups in the quantitative phase (faculty and students in pilot implementations). In all cases, non-probabilistic purposive convenience sampling was used. This criterion is appropriate for exploratory mixed methods designs as it allows for the selection of key informants and ensures disciplinary diversity.

#### 2.2.1 Qualitative phase

##### 2.2.1.1 Interviews with faculty

Fourteen faculty members from three Chilean universities participated in the interviews. All participants taught first- or second-year courses using virtual classrooms based on LMS. The sample comprised six men and eight women from different disciplinary areas, such as physical and mathematical sciences, chemical sciences, social sciences, education, and humanities. Table 1 presents the frequency of faculty members by gender and disciplinary area.

**Table 1:** Frequency of faculty members by gender and disciplinary area.

Disciplinary area	Faculty members	Men	Women
Physical and mathematical sciences	2	2	0
Chemical sciences	2	0	2
Biological sciences	1	1	0
Engineering	1	1	0
Social sciences	3	0	3
Education	1	1	0
Health sciences	2	0	2
Humanities	1	0	1
Architecture	1	1	0
Total	14	6	8

##### 2.2.1.2 Student focus groups

A total of 19 focus groups were conducted with 140 students (88 women, 52 men) aged 20 to 30 years (*M* = 21.53, SD = 2.87). Participants were enrolled in courses taught by the previously interviewed faculty members or by five other academics who facilitated recruitment. The students represented several disciplinary areas, including physical and mathematical sciences, humanities and arts, engineering, social sciences, health sciences, and education.

##### 2.2.1.3 Expert review

Seven academics participated, selected via chain or “snowball” sampling. All participants held doctoral degrees and had experience in educational technology in higher education. The sample comprised five women and two men from disciplinary areas including social sciences, basic sciences, engineering, health, and education.

#### 2.2.2 Quantitative phase

##### 2.2.2.1 Pilot implementations (1 and 2)

Faculty members were invited from one of the participating universities who taught first-year courses identified as high-risk due to their high failure rates. The selection process focused on faculty members whose virtual classrooms demonstrated high levels of activity and who utilized a diverse range of learning resources. The aim was to recruit one instructor from each school. Of these, five faculty members agreed to participate by integrating the SRL app into one of their virtual classrooms (see Table 2).

**Table 2:** Frequency of faculty members and student participants in the pilot implementation.

Disciplinary area	Faculty members	Students
Physical and mathematical sciences	1 (W)	69
Biological sciences	1 (W)	9
Chemical sciences	1 (M)	69
Engineering	1 (M)	29
Social sciences/ Health sciences	1 (W)	70
Total	5	246

W = woman and M = man.

The second implementation was conducted using a usability-refined version of the tool that retained the same core functionalities evaluated in Pilot 1. The refinements concerned the interface and user experience rather than the functional architecture, including adjustments to visual presentation, element positioning, screen organization, visual hierarchy, and navigation intended to make the application more intuitive and user-friendly. The study took place in a single course taught by an instructor from the initial pilot who had expressed interest in continuing to use the app. The course was a first-year Basic Sciences class composed of 21 students (13 men and 8 women). Given its small sample size and single-course scope, Pilot 2 is best characterized as a small-scale usability and feasibility pilot rather than a study powered to detect stable between-group differences.

### 2.3 Instruments

#### 2.3.1 Semi-structured interviews and focus groups

A semi-structured interview and focus group protocol was developed by the research team for both the faculty interviews and the student focus groups. This protocol covered three main topics: (a) the conceptualization of SRL in BL contexts, (b) SRL strategies used by students in BL settings, and (c) facilitators and barriers to SRL in these environments. The semi-structured nature of the protocol allowed us to maintain a common thematic focus while providing the flexibility to explore emergent themes from participant responses.

Interviews and focus groups were conducted in Spanish, the participants’ native language, and lasted between 60 and 90 min. Focus groups comprised between 7 and 10 students in most cases, with one group of 5 students (140 students across 19 groups overall; see Table 3). The sessions were audio recorded using a mobile phone voice recorder and subsequently transcribed verbatim using the application https://www.amberscript.com; after analysis, members of the research team with English proficiency translated representative quotes for presentation in this manuscript.

**Table 3:** Frequency of student focus group participants by gender and disciplinary area.

Disciplinary area	Number of focus groups	Men	Women	Total
Physical and Mathematical sciences	6	17	9	26
Humanities and arts	2	3	17	20
Engineering	3	18	4	22
Social sciences	4	10	30	40
Health sciences	2	4	8	12
Education	2	0	20	20
Total	19	52	88	140

#### 2.3.2 Expert review

To assess the functional relevance of the application, a checklist was developed based on the three phases of SRL and their respective strategies. Expert reviewers rated each of the app functionalities on a three-point achievement scale: Achieved, Partially Achieved, or Not Achieved. For functionalities rated in the latter two categories, a written justification was requested with qualitative feedback aimed at improving the design. To ensure a comprehensive evaluation, the research team monitored each expert’s usage analytics and prompted any reviewer who had not adequately explored a feature before submitting their rating.

The checklist included operational definitions for each SRL dimension and subdimension represented in the application, providing experts with a common conceptual reference for judging the extent to which each functionality addressed the intended self-regulatory strategy. It also included a progression rubric specifying the criteria for assigning each rating: Achieved indicated that the functionality adequately represented and supported the intended SRL strategy; Partially Achieved indicated that the functionality was present and aligned with the strategy but required refinement, greater depth, or improved implementation; and Not Achieved indicated that the intended strategy was absent or insufficiently represented by the functionality.

Experts completed the checklist independently, and their individual ratings were retained as reported in Table 4. Inter-expert agreement was computed *post hoc* from these ratings using percentage agreement and Fleiss’ kappa. Mean item-level percentage agreement across the 10 SRL strategies was 84.3%, and Fleiss’ kappa was 0.60, indicating moderate-to-substantial agreement (Landis and Koch, 1977). Agreement was highest (100%) for functionalities in the planning phase related to study-space preparation, digital material organization, time management, and external benchmark monitoring, and lowest for social learning strategies (57.1% agreement; 4/7 Achieved, 3/7 Partially Achieved) and for the self-reflection strategies of learning intentions, self-judgment, and self-motivation (71.4% agreement each), consistent with the comparatively lower ratings reported for the self-reflection phase.

**Table 4:** Expert judgment on the functionalities of the self-regulated learning application (Regula2).

Stage	Strategies	J1	J2	J3	J4	J5	J6	J7
Planning	Time management	PA	PA	PA	PA	PA	PA	PA
	Preparation of the study space	A	A	A	A	A	A	A
	Preparation of digital materials and resources	A	A	A	A	A	A	A
	Study strategy	PA	PA	PA	PA	A	A	PA
	Learning intentions	NA	PA	NA	NA	NA	NA	PA
Performance	External benchmarks for self-monitoring	PA	PA	PA	PA	PA	PA	PA
	Personal learning strategies	PA	PA	PA	PA	PA	PA	PA
	Social learning strategies	A	PA	PA	A	A	A	PA
Self-reflection	Self-judgment	NA	NA	NA	NA	NA	PA	PA
	Self-motivation	NA	PA	NA	NA	NA	NA	A

A = Achieved; PA = Partially Achieved; NA = Not Achieved. J1–J7 = Judges 1–7.

#### 2.3.3 Scale for perceived impact and technological acceptability of the SRL app

In the pilot implementations, students responded to a five-point Likert-type scale (1 = Strongly Disagree, 5 = Strongly Agree) developed *ad hoc* for this study. The instrument comprised three dimensions corresponding to the phases of SRL: Forethought (7 items), Performance (6 items), and Self-Reflection (5 items). Item-level descriptive statistics are provided in full in the Supplementary material. Additionally, two items inspired by the Technology Acceptance Model (TAM; Davis, 1989) were included to measure the app’s perceived ease of use and perceived usefulness. An example item from the scale is “The app helped me better organize my study time.” Internal consistency of the three *ad hoc* SRL subscales was assessed using data from Pilot 1 was acceptable to good: Forethought (*α* = 0.87, ω = 0.89), Performance (*α* = 0.81, ω = 0.82), and Self-Reflection (*α* = 0.84, ω = 0.91). Perceived usefulness and perceived ease of use were each measured with a single item, consistent with the abbreviated TAM format used in related SRL technology studies. The 18 SRL items were developed *ad hoc* by the research team, drawing directly on the qualitative challenge and strategy taxonomy (Tables 5, 6) and organized according to Zimmerman’s three-phase model; they were not adapted from a pre-existing validated questionnaire. Formal cognitive interviews or item comprehensibility testing were not conducted prior to administration; instead, the two pilot implementations themselves served as the primary empirical check on how the items functioned in practice, consistent with the iterative, exploratory nature of this design.

**Table 5:** SRL challenges in blended learning, taxonomy with representative participant quotations.

Category	Subcategory	Representative quotation
Organization	Digital resources	“I think your level of personal organization really matters. You need to have everything handy, whether it’s in the cloud or on the course platform; otherwise, you waste time and just cannot keep up” (FG16). “Everything has to be in labeled folders so you do not lose track of it, because trying to find things later... ugh, it’s a nightmare” (FG8).
	Study spaces	“A stable internet connection is essential” (FI3). “A quiet physical space without distractions, where you will not be interrupted” (FG7).
Academic workload	Quantity of learning resources	“I feel like the virtual classrooms are bombarded with too many files and documents; it’s overwhelming” (FI4).
	Prioritizing learning resources	“If you do not know what’s important, you get lost. There is so much material available, but you cannot review it all” (FG10).
Learning strategies	Strategies for selecting learning materials	“In the virtual setting, you do not just consume content by downloading it; you have to know how to select it, organize it, and actually study it” (FG19).
	Visual learning strategies	“I realized that with summaries I could remember parts, but with mind maps I understood how the topics were connected” (FG9). “I made a comparative chart, and that’s when I finally understood the differences” (FG6).
Academic interaction	Limited communication opportunities	“In person, I can ask the professor if I have questions, but not in the virtual setting” (FG2). “You do not know who to ask, so you start searching on the internet” (FG12).
	Lack of clear benchmarks	“You do not know if you are doing it right. At least in class, you can look at others and realize when you are messing up” (FG4).
Feedback	Infrequent feedback	“There are fewer opportunities for feedback... so you lose that feedback loop” (FI2).
	Delayed feedback	“In the physical classroom, you can ask the professor if you are on the right track. When you submit assignments in the virtual classroom, you just hand them in, and that’s it” (FI11).
Self-care habits	Meal schedules	“You’re supposed to stick to a schedule, but sometimes I just work straight through and realize I have not eaten anything at all” (FG4).
	Sleep schedules	“You lose track of time, and you either do not sleep or you sleep very little” (FG6).
Motivation	External motivation	“I do not understand the material, and it’s like I just crash and then do not want to do anything anymore” (FG2). “The only way to get them to engage with the virtual classroom is if it counts towards their grade” (FI1).

FG = Focus Group participant; FI = Faculty Interview participant.

**Table 6:** SRL strategies in blended learning, taxonomy with representative participant quotations.

Category	Subcategory	Representative quotation
Planning	Integrated calendars	“I wish everything (academic commitments) was in one place, so you did not have to click a million times” (FG3). “You have to organize your life, schedule everything, the virtual, the in-person, all of it” (FG15).
Estimation of cognitive resources	Task complexity	“When I first saw the instructions, I thought I just had to read a text, but then I realized I had to connect it with other readings and apply a formula... so it was much more complex than it seemed at first” (FG16). “This algebra topic is harder for me because it’s not just about applying steps; you have to really understand the reasoning behind it” (FG2).
	Effort estimation	“To understand this module, I had to read it twice, highlight, summarize, and find a separate video... I realized it was going to take a lot of effort, so I mentally prepared myself” (FG10).
	Time estimation	“At first, I thought the activity would take me an hour, but when I started reviewing the materials, I realized there was a lot, so it ended up taking me the whole afternoon” (FG10).
Social co-regulation	Peer feedback	“In the forum, I commented on a classmate’s essay, and later she gave me feedback on mine... it helped me better organize my ideas for the next activity” (FG18).
	Peer recognition	“In our Teams group, after the presentation, several people wrote to me saying that it had been super clear. That was really motivating because with the cameras off, you never know if you are doing a good job” (FG12).
	Identifying peers’ strengths	“When we have doubts, we always ask Seba in the WhatsApp group because he’s amazing at finding videos or tutorials that explain things better than the official guide” (FG8).
Instrumental collaboration	Sharing learning materials	“Vale uploaded her summary of the chapter to the WhatsApp group. It helped me a lot because it was much clearer than the guide they uploaded to the platform” (FG11).
	Sharing external resources	“A classmate sent a YouTube video that explained the material better, even better than the professor” (FG4).
	Modeling	“I saw a classmate’s presentation and realized she had used images with people in them, and I had been unintentionally violating ethical standards, so I’ll never do that again” (FG14).
	Metacognition	“After reading the comments another classmate received, I started to think about whether I was also articulating my ideas well, and I ended up fixing my work” (FG10).
Use of benchmarks	Performance benchmarks	“When I saw the professor’s comments to others in the forum, I realized that I was also explaining things without justifying them with scientific evidence” (FG6). “They see that most of their classmates have already submitted the assignment, so they hurry up” (FI2).
	Grade-based benchmarks	“I scored lower than the class average on the quiz, so I knew something was wrong with how I was studying. That’s why I started using flashcards instead of just reading.”
Activation of motivation	Micro-goals	“I learned to be realistic and to focus. For example, today I’ll only focus on doing the first two exercises, tomorrow two more” (FG3).
	Rewards	“After finishing the test, I took an hour to watch a series. I earned it.” (FG6).
	Positive self-talk	“I tell myself: this is difficult, but I’ve solved more complex things before” (FG1). “I give myself encouragement: you can do this, come on, you are almost there” (FG3).
Maintenance of motivation	Managing negative thoughts	“You have to control your thoughts, the ones that tell you that you cannot do it, the ones that bring you down” (FG2).
	Judgments about performance	“You have to be self-critical and see what you did well and what you did not” (FG5).

FG = Focus Group participant; FI = Faculty Interview participant.

### 2.4 Procedure

The study unfolded across four sequential stages. In Stage 1, faculty were contacted by email and invited to participate in individual interviews and to facilitate student recruitment for focus groups. Focus groups were held on-site at each university, with voluntary registration managed through an online form. All sessions were audio recorded. Verbatim transcripts were produced for all interviews and focus groups prior to analysis.

In Stage 2, the expert review was implemented. Experts received access to the preliminary app prototype, explored its features independently, and completed the checklist, providing both quantitative ratings and written qualitative justifications that informed targeted design revisions. Expert engagement with each feature was verified through usage analytics before ratings were accepted.

Stage 3 (Pilot 1) began with an informational meeting for participating instructors, followed by a three-session training workshop covering (a) SRL theoretical foundations, (b) student-perspective app navigation, and (c) integration of the app into a course learning activity. Students used the app during the implementation period (spanning the full course semester) and completed the scale immediately afterwards. Following Pilot 1, the core functionalities of Regula2 were retained, while usability-oriented interface refinements were introduced to improve visual organization, navigation, and ease of interaction. Stage 4 (Pilot 2) deployed this interface-refined version in a single course following the same training and implementation protocol. The same scale was administered, allowing a descriptive juxtaposition of ratings across two non-equivalent implementations. Across both pilot implementations combined, platform analytics recorded a total of 1,932 student logins over the implementation period, corresponding to an average of approximately 7.2 logins per student.

### 2.5 Ethical considerations

This study was conducted in accordance with the ethical principles of the Declaration of Helsinki and applicable Chilean research ethics regulations. All procedures involving human participants were approved by the Institutional Ethics Committee of Universidad de Concepcion (Protocol No. CEBB 1099–2022). Voluntary participation and informed consent were obtained from all participants across all stages. Participants were briefed on the study’s purpose, data use, and confidentiality protections. All data were stored in secure institutional databases and used exclusively for research purposes. No data were collected from minors; all participants were enrolled university students aged 18 years or older.

### 2.6 Data analysis

Qualitative data from interviews and focus groups were analyzed using qualitative content analysis with an inductive orientation. The unit of coding was the unit of meaning, defined as a word, phrase, sentence, or set of sentences expressing a relevant idea in relation to the study aims. Following comprehensive reading and familiarization with the corpus, open coding was conducted and codes were iteratively grouped into higher-order thematic categories. Faculty and student data were integrated into a common analytic corpus because the purpose was to understand a shared phenomenon rather than to formally compare participant groups; however, the source of each excerpt was retained throughout coding so that group-specific nuances or divergent perspectives could be identified when analytically relevant. Coding was carried out collaboratively by two researchers, who discussed units of meaning, codes, and category boundaries throughout the analysis and resolved interpretive differences through consensus. Because coding was not performed independently, no inter-coder agreement coefficient or Cohen’s kappa was calculated. Negative cases and divergent opinions were retained and considered during category development to qualify predominant patterns rather than being excluded from the analysis. Recurrence, consistency, and diversity of perspectives across the corpus were used to assess thematic sufficiency; saturation was treated as an analytic adequacy criterion rather than as a formal stopping rule for recruitment. Sessions were transcribed verbatim from audio recordings, and the interpretation of the data, including the extraction of units of meaning and the development of codes, categories, and dimensions was carried out manually using spreadsheet software (Microsoft Excel).

Quantitative pilot data were examined using descriptive statistics (means and standard deviations by scale dimension and item). No inferential comparisons between pilots were conducted, given differences in sample size and course context. The between-pilot contrast was therefore treated as observational and descriptive. Because the core functional architecture was preserved between Pilot 1 and Pilot 2, higher ratings in Pilot 2 were interpreted as being consistent with a more usable and accessible interface rather than as evidence that new functionalities caused improvements in perceived impact.

## 3 Results

Results are organized across four sequential stages: (a) characterization of SRL challenges and strategies in blended learning contexts, (b) preliminary tool design, (c) expert review and iterative refinement, and (d) perceived impact and technological acceptability across two pilot implementations.

### 3.1 Qualitative results

#### 3.1.1 Characterization of SRL in blended learning contexts

Challenges. Content analysis of faculty interviews and student focus groups identified five overarching challenge domains in BL contexts. The seven categories shown in Table 5 were synthesized into these five domains for narrative discussion: Organization; Information overload, encompassing academic workload and learning-strategy challenges; Academic interaction; Feedback; and Motivation. Self-care disruption, the seventh category in Table 5, is discussed separately below as a cross-cutting factor rather than as one of the five primary domains. Table 5 presents the full taxonomy with representative participant quotations.

Organization emerged as a foundational difficulty, the students described the need to keep course materials classified and instantly accessible across platforms, with disorganized digital environments producing wasted time and loss of learning continuity. Closely related was information overload the abundance of files in virtual classrooms left students unable to prioritize relevant resources without instructor guidance, generating feelings of overwhelm. A third cluster involved limited academic interaction: reduced opportunities for synchronous contact with instructors and peers created uncertainty about task correctness and eroded access to timely feedback, which students described as infrequent and delayed relative to face-to-face contexts. Motivational regulation was a fourth domain, particularly when virtual content was decoupled from assessment or when difficulty was encountered without immediate support, conditions that triggered disengagement and task abandonment. Finally, self-care disruption (irregular sleep and meal schedules) was identified as an indirect but recurrent factor affecting self-regulatory capacity, often exacerbated by the absence of clear temporal boundaries in online work.

Strategies. In parallel, participants reported an active repertoire of compensatory SRL strategies, organized into four dimensions. Table 6 presents the full strategy taxonomy with representative quotations.

Individual planning centered on integrated calendar use to coordinate virtual, in-person, and personal academic commitments simultaneously. Cognitive resource estimation involved students appraising task complexity, required effort, and time demands before engaging, a metacognitive practice enacted informally but consistently, as illustrated in Table 6. Social co-regulation was prominent: students shared materials, exchanged external resources, identified peers with complementary organizational strengths, and used peer modeling to calibrate their own study approaches and activate metacognitive comparison. Benchmark-based self-monitoring involved contrasting individual performance against peer behavior, grade averages, and instructor-visible forum contributions to inform corrective decisions. Motivational self-regulation strategies (micro-goals, self-reward, positive self-talk, and negative-thought management) were reported as essential for sustaining effort in low-feedback virtual environments. Collectively, these strategy clusters reveal that students in BL contexts engage in sophisticated informal regulatory activity that technology design should scaffold rather than displace.

### 3.2 Preliminary tool design

Drawing on the qualitative findings and Zimmerman's (2002) three-phase SRL model, we specified the functional architecture of Regula2. The forethought phase was addressed through time management, cognitive load estimation (complexity, effort, time), study space preparation, learning material organization, and learning intention setting. The performance phase was addressed through external benchmark visualization, personal strategy monitoring, and peer collaboration features. The self-reflection phase, identified as the most underserved in literature, was targeted through post-activity estimation comparison and motivational gamification. The initial prototype comprised seven interfaces developed iteratively through weekly design sprints between the research and development teams, with user interface decisions validated at each sprint. This co-design process ensured that architectural decisions remained grounded in the challenge and strategy taxonomy documented in Stage 1, rather than in *a priori* assumptions about student regulatory behavior.

### 3.3 Expert review and iterative refinement

Seven doctoral-level experts evaluated each of the 10 SRL strategy areas across the three Zimmerman phases using the three-point checklist (Table 4). Results showed clear differentiation by SRL phase. Mean item-level percentage agreement across strategies was 84.3%, and Fleiss’ kappa was 0.60, indicating moderate-to-substantial inter-expert agreement (Landis and Koch, 1977).

In the forethought phase, study space preparation and digital material organization were unanimously rated Achieved, indicating adequate design coverage. Time management and study strategy selection were rated Partially Achieved by the majority, reflecting functional presence but insufficient depth. Learning intentions was predominantly rated Not Achieved (5/7 reviewers), identifying goal meaning and utility as a critical unresolved gap, reviewers noted that the preliminary interface prompted intention entry but provided no scaffolding for goal formulation quality.

In the performance phase, both external benchmark monitoring and personal strategy monitoring received unanimous Partially Achieved ratings, pointing to adequate but optimizable progress-tracking features. Social learning strategies showed more heterogeneous ratings (Achieved to Partially Achieved), reflecting variability in how fluidly peer collaboration features were accessed.

The self-reflection phase received the lowest ratings overall: self-judgment and self-motivation were predominantly rated Not Achieved (5–6/7 reviewers), confirming this phase as the primary design gap consistent with the qualitative findings and prior literature on the difficulty of supporting metacognitive closure in LMS-integrated tools (Edisherashvili et al., 2022; Pinheiro et al., 2021). Reviewers specifically noted the absence of structured self-evaluation prompts and the lack of any mechanism for students to compare intended with actual learning outcomes.

Expert feedback drove targeted functional revisions before the pilot implementations. In the forethought phase, we expanded the calendar to include personal events alongside LMS-sourced commitments, added a weekly view for granular planning, introduced checkable task lists, and implemented a cognitive load estimation feature comparing pre-activity expectations with post-activity experience. A study strategy repository indexed by learning objective was incorporated. In the performance phase, push notifications on personal and course-level progress were added, alongside pop-up self-monitoring reminders and improved visual distinction between active and completed events; a dedicated help-request button strengthened peer collaboration access. For self-reflection, a gamification system with a participation leaderboard was introduced to provide social recognition of effort — a partial but feasible response to the unresolved self-judgment gap. Two self-reflection features (goal attainment verification and structured performance reflection space) could not be resolved in the current version and represent priorities for future development. These expert-informed revisions established the functional architecture subsequently evaluated in Pilot 1; no additional core functions were added or removed between Pilot 1 and Pilot 2.

### 3.4 Final app design

The final version of Regula2 comprises seven interfaces organized according to the three phases of Zimmerman’s SRL model. Figures 1–3 present the interfaces grouped by the SRL phase they primarily support, illustrating the structural coverage and design rationale of each functional area. The core functional architecture described below was already present in Pilot 1 and was retained in Pilot 2; the changes introduced between the two pilots were limited to usability-oriented refinements of the interface and user experience rather than changes in functionality.

**Figure 1:**
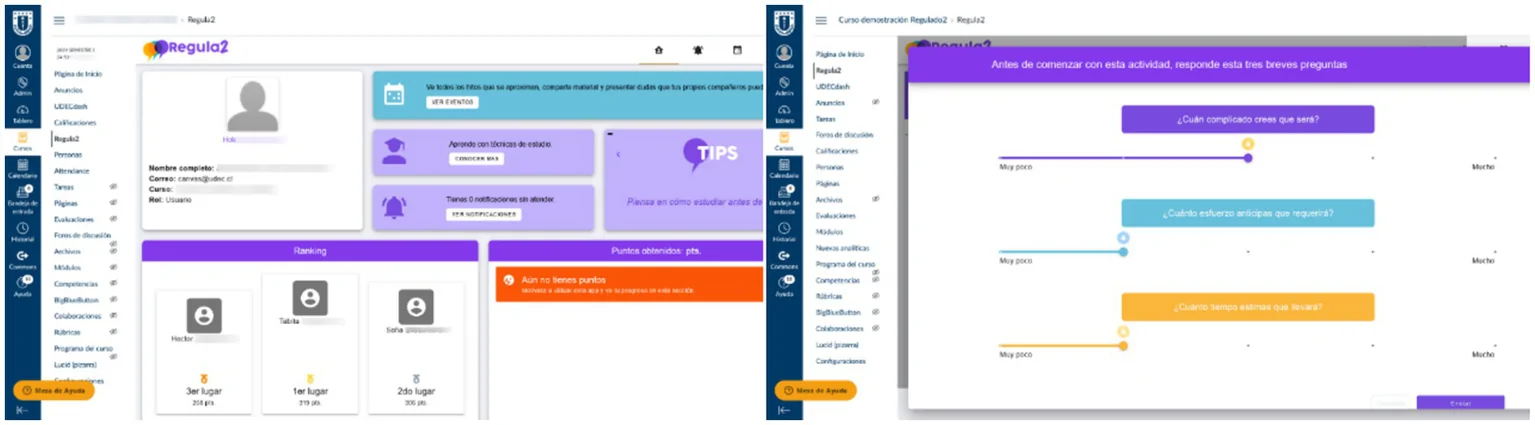
Forethought phase—Interface 1 (left): Main dashboard centralizing upcoming LMS events, study technique recommendations, push notifications, and a gamification ranking derived from student interactions. Interface 2 (right): Cognitive load estimation module. Before engaging in a course event, students respond to three Likert-type sliders estimating task complexity, required effort, and expected time. Responses are stored for post-activity comparison.

**Figure 2:**
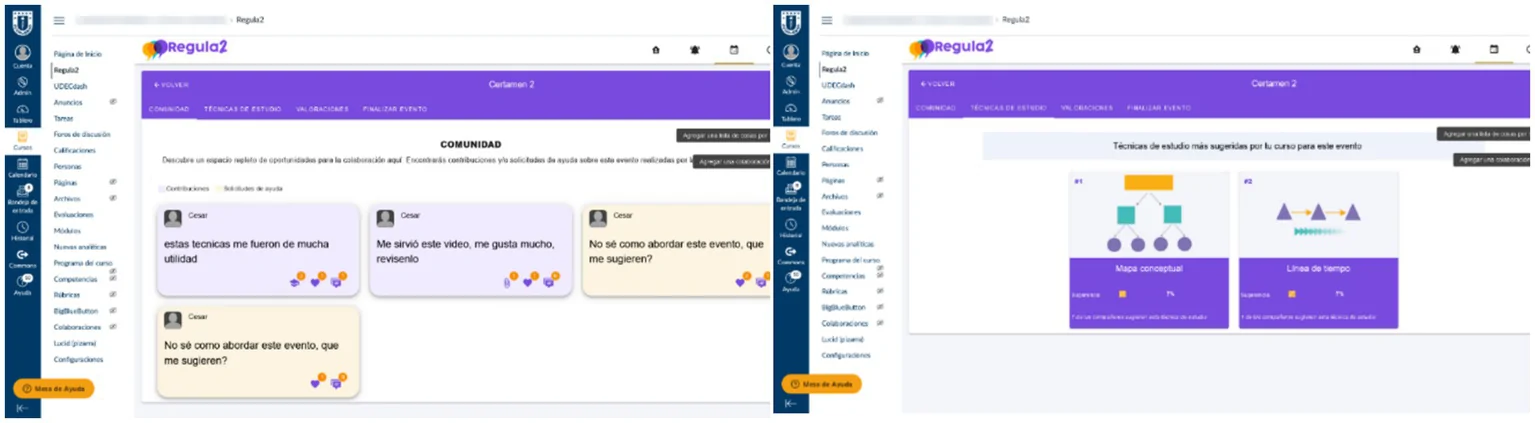
Performance phase—Interface 3 (left): Community space enabling students to share supplementary materials, experiences, and help requests linked to specific course events. Contributions support peer modelling and social co-regulation. Interface 4 (right): Study techniques aggregator displaying a ranked summary of peer-proposed strategies for a given event, operationalizing benchmark-based self-monitoring through class-level comparison.

**Figure 3:**
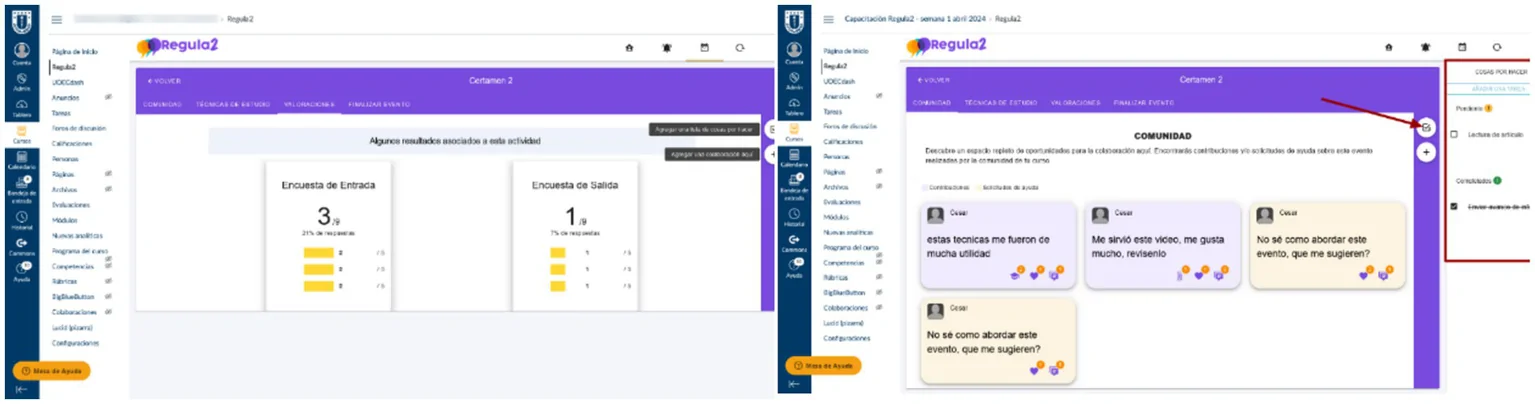
Self-reflection phase—Interface 5–6 (left): Assessments and to-do panel. Interface 5 displays aggregated entry/exit estimation results at course level, enabling comparison of cognitive load predictions with actual experience. Interface 6 provides a personal task checklist supporting granular progress tracking. Interface 7 (right): Community contribution forum with integrated to-do panel. Each community contribution has an associated discussion thread for peer feedback. The task panel (right side) tracks pending and completed sub-tasks, supporting self-monitoring and metacognitive closure.

Forethought phase (planning). Interface 1 (Figure 1, left) provides the main dashboard, centralizing upcoming LMS events, study technique recommendations, push notifications, and a gamification ranking derived from student interactions. Interface 2 (Figure 1, right) houses the cognitive load estimation module: before engaging with a course event, students respond to three Likert-type sliders estimating task complexity, required effort, and expected time. Responses are stored for post-activity comparison in the self-reflection phase, directly operationalizing the metacognitive calibration identified in the qualitative strategy taxonomy (Table 6).

Performance phase (monitoring and collaboration). Interface 3 (Figure 2, left) provides a community space enabling students to share supplementary materials, experiences, and help requests linked to specific course events. Contributions support peer modeling and social co-regulation, directly extending the social strategy cluster identified in Stage 1. Interface 4 (Figure 2, right) is a study techniques aggregator displaying a ranked summary of peer-proposed strategies for a given event, operationalizing benchmark-based self-monitoring through class-level comparison.

Self-reflection phase (evaluation and self-motivation). Interfaces 5–6 (Figure 3, left) form the assessments and to-do panel. Interface 5 displays aggregated entry/exit estimation results at course level, enabling students to compare cognitive load predictions with actual experience — directly responding to the expert-identified self-judgment gap. Interface 6 provides a personal task checklist supporting granular progress tracking. Interface 7 (Figure 3, right) integrates a community contribution forum with a to-do panel: each community contribution has an associated discussion thread for peer feedback, and the task panel tracks pending and completed sub-tasks, supporting self-monitoring and metacognitive closure.

To make the integration of the qualitative and quantitative strands of this exploratory sequential design more explicit, Table 7 traces how qualitative needs identified in Stage 1 informed Regula2 functions, how expert review led to functional refinements before Pilot 1, and how Pilot 1 ratings subsequently informed usability-oriented UX/UI refinements while preserving the same core functions for Pilot 2. Pilot 2 ratings are then interpreted in relation to these earlier qualitative requirements and design decisions.

**Table 7:** Integration of qualitative needs, expert-informed functional refinement, Pilot 1 evidence, UX/UI refinement, and Pilot 2 perceived-impact ratings across the three SRL phases.

Qualitative need (Stage 1)	SRL phase	Function informed by qualitative findings	Expert review and functional refinement before Pilot 1	Pilot 1 evidence and UX/UI refinement	Pilot 2 perception and integrative interpretation
Difficulty organizing time, activities, and materials across virtual and in-person commitments; need for integrated calendars (Tables 5, 6)	Forethought	Time management, study space preparation, material organization, learning intention setting, and cognitive resource estimation.	Time management and study strategy were rated Partially Achieved by most experts, and learning intentions were rated Not Achieved by 5/7. Before Pilot 1, the calendar was expanded, a weekly view and checkable task lists were added, cognitive load estimation with pre/post comparison was implemented, and a study-strategy repository was incorporated.	Pilot 1 forethought ratings were low to moderate (*M* = 2.56–2.86). The core functions were retained for Pilot 2; subsequent changes focused on interface presentation, visual hierarchy, element positioning, screen organization, and navigation to make the same functions more intuitive and user-friendly.	Pilot 2 forethought ratings ranged from *M* = 4.0–4.8; activity organization (*M* = 4.8) and time management (*M* = 4.5) were highest. Because functionality was unchanged, the descriptively higher ratings are consistent with improved access to and interaction with the existing planning functions rather than the addition of new functions.
Need for peer feedback, resource sharing, and benchmark-based self-monitoring against classmates (Table 6)	Performance	External benchmark visualization, personal strategy monitoring, peer collaboration, resource sharing, and help-seeking functions.	External benchmark and personal strategy monitoring were unanimously rated Partially Achieved; social learning strategies were rated Achieved to Partially Achieved. Before Pilot 1, progress notifications, self-monitoring reminders, improved distinction between active/completed events, and a dedicated help-request button were incorporated.	Pilot 1 performance ratings ranged from *M* = 2.63–2.98. The same performance functions were retained for Pilot 2, while their visual presentation, positioning, and navigation were refined to support easier interaction.	Pilot 2 collaboration and resource-sharing items reached *M* = 4.2–4.4, while self-monitoring remained lower (*M* = 3.8–3.9). The pattern supports the continued relevance of the qualitative requirement for social co-regulation, while indicating that individual self-monitoring remained comparatively less well supported.
Limited opportunity for self-evaluation and closing the feedback loop after academic activities (Tables 5, 6)	Self-reflection	Post-activity estimation comparison and motivational gamification.	Self-judgment and self-motivation were predominantly rated Not Achieved (5–6/7), identifying self-reflection as the main functional gap. Before Pilot 1, pre/post cognitive-load comparison and gamification/participation ranking were incorporated; goal-attainment verification and a structured reflection space remained unresolved.	Pilot 1 self-reflection ratings were the lowest (*M* = 2.23–2.37). The same self-reflection functions were retained for Pilot 2; changes between pilots were limited to UX/UI refinements intended to make access and interaction more intuitive.	Pilot 2 estimation-comparison items reached *M* = 4.4, whereas holistic self-evaluation remained more moderate (*M* = 3.7–3.8). The integrated evidence suggests that structured calibration was better supported than broader self-judgment, which remained an unresolved design need.

### 3.5 Perceived impact and technological acceptability

Results from the two pilot implementations are descriptively juxtaposed as part of the cross-stage integration of the exploratory sequential design. Pilot 1 assessed the expert-informed functional version of Regula2. Following Pilot 1, the core functions were preserved, while the interface underwent usability-oriented refinements intended to make navigation and interaction more intuitive and user-friendly. Accordingly, Pilot 2 ratings are interpreted as descriptive evidence of students’ perceptions of the same functional architecture presented through a refined user interface, rather than as evidence of the effect of newly added functions. Item-level descriptive statistics for both pilots are provided in full in the Supplementary material.

#### 3.5.1 Pilot 1: preliminary version

Across all three SRL phases, students reported low-to-moderate perceived experiences, with item means consistently in the range of *M* = 2.23–3.21. The highest scores were associated with features supporting peer resource exchange (performance phase; *M* approximately 2.88–2.98) and perceived usefulness overall (*M* = 3.21, SD = 1.59). The lowest scores appeared in the self-reflection phase, particularly for evaluating one’s learning process after task completion (M = 2.23, SD = 1.25) and identifying learning level achieved (*M* = 2.30, SD = 1.28), findings that directly paralleled the expert review results and confirmed the self-reflection gap as the most urgent design priority.

In the forethought phase, items related to time organization (*M* = 2.56, SD = 1.40) and activity organization (*M* = 2.58, SD = 1.40) received the lowest scores alongside study strategy anticipation (*M* = 2.65, SD = 1.45). Cognitive load estimation items — estimating complexity (*M* = 2.72, SD = 1.48), time (*M* = 2.74, SD = 1.50), and effort (*M* = 2.86, SD = 1.53) were marginally higher, which may indicate that these functions were perceived as relevant even where the preliminary interface did not fully support them, though this interpretation remains tentative given the descriptive nature of the data. In the performance phase, resource sharing and peer collaboration items clustered around *M* = 2.88–2.98, while self-monitoring items (*M* = 2.77, SD = 1.54) remained lower.

#### 3.5.2 Pilot 2: final version

Following Pilot 1, no core functional components were added to or removed from Regula2. Instead, the application underwent a usability-oriented interface refinement, including adjustments to visual presentation, element positioning, screen organization, visual hierarchy, and navigation, with the aim of making the existing functions more intuitive and user-friendly. Perceived impact ratings in Pilot 2 were descriptively higher across all three SRL phases. In the forethought phase, item means ranged from *M* = 4.0–4.8 (SD = 0.5–1.2), with activity organization (*M* = 4.8, SD = 0.5) and time management (*M* = 4.5, SD = 0.7) as the highest-rated items — compared to *M* = 2.56–2.86 in Pilot 1, a descriptive difference of approximately 1.9–2.2 points. Study strategy anticipation (*M* = 4.1, SD = 0.9), cognitive complexity estimation (*M* = 4.0, SD = 0.8), time estimation (*M* = 4.1, SD = 0.9), and effort estimation (*M* = 4.0, SD = 0.9) were also descriptively higher. Given the small sample size, item-level medians, interquartile ranges, and full response-frequency distributions (percentage of students selecting each scale point) are also provided alongside means in the Supplementary material.

In the performance phase, resource-sharing and peer collaboration items reached *M* = 4.2–4.4 (SD = 0.7): exchanging resources with classmates (*M* = 4.4, SD = 0.7), sharing important materials (*M* = 4.4, SD = 0.7), and asking classmates for help (*M* = 4.2, SD = 0.7) were the highest-rated items. Self-monitoring items were descriptively higher than in Pilot 1 but remained comparatively lower within Pilot 2: monitoring learning processes (*M* = 3.9, SD = 1.1) and evaluating study strategy effectiveness (*M* = 3.8, SD = 1.2). This pattern suggests that, even with the refined interface, individual self-monitoring remained more challenging than peer-oriented collaboration and resource sharing.

In the self-reflection phase, the estimation-comparison features achieved the highest scores in this dimension: complexity comparison (*M* = 4.4, SD = 0.6), effort comparison (*M* = 4.4, SD = 0.7), and time comparison (*M* = 4.4, SD = 0.6), compared to *M* = 2.33–2.37 in Pilot 1, a descriptive difference of approximately 2.0–2.1 points. These functions were already present in Pilot 1 as part of the expert-informed functional architecture and were retained unchanged in Pilot 2. Thus, the higher Pilot 2 ratings do not reflect the addition of a new cognitive load comparison module; rather, they are consistent with the possibility that the refined interface facilitated students’ access to and interaction with this existing structured prompt for reflective contrast. Holistic learning evaluation items remained more moderate: evaluating the learning process (*M* = 3.8, SD = 1.3) and identifying learning level achieved (*M* = 3.7, SD = 1.5), consistent with the structural limitations in self-judgment scaffolding identified in expert review.

TAM items were also descriptively higher in Pilot 2: perceived usefulness increased from *M* = 3.21 (SD = 1.59) to *M* = 4.1 (SD = 1.2), and perceived ease of use from *M* = 2.86 (SD = 1.55) to *M* = 3.8 (SD = 1.2). Because the core functionality remained unchanged between pilots, this pattern is consistent with, but does not establish, improved usability and accessibility following the UX/UI refinements. In Pilot 2, perceived usefulness exceeded perceived ease of use (4.1 vs. 3.8), a configuration generally considered favorable for adoption in the TAM literature.

## 4 Discussion

### 4.1 Characterization of SRL in blended learning contexts

#### 4.1.1 Challenges of SRL in blended learning contexts

Our findings show that the challenges for SRL in BL environments reported by faculty and students were not framed solely as a matter of individual skill but were consistently attributed to contextual and structural factors. This pattern is consistent with Faza and Lestari (2025) and Lobos et al. (2024), who similarly describe factors ranging from digital distractions and limited pedagogical guidance to technological design and institutional infrastructure as mediators of students’ capacity to plan, monitor, and evaluate their own learning. In our data, personal organization, materials management, and the availability of adequate physical and virtual spaces emerged as the most salient of these elements. The overload of resources in virtual classrooms and the absence of clear criteria for prioritizing them, reported by students in our focus groups, align with prior findings that information saturation is an obstacle to effective planning (Aristovnik et al., 2020; Lobos et al., 2021a).

This experience of cognitive overload is also consistent with neuroscientific accounts of stimulus-dense digital environments, which link an abundance of resources without clear management criteria to mental fatigue and reduced sustained attention (Bobrytska et al., 2025). Similarly, the limitations in academic interaction and timely feedback that our participants described as eroding their capacity to monitor and adjust study strategies echo evidence that LA-based feedback can support self-regulated learning and academic performance by delivering timely, decision-relevant information (Chen, 2024) — suggesting that the feedback gap identified by our participants reflects a broader pattern documented elsewhere rather than a feature specific to this sample.

In the same vein, our finding that students described disengagement when support was inconsistent is consistent with Heikkinen et al. (2025), who report that self-regulatory skill development and student engagement progress more robustly under continuous support; conversely, its absence appears to slow or inhibit these processes, an effect our participants described as especially pronounced among those with lower initial engagement.

Finally, the self-care disruption and motivational strain reported by our participants reinforce the view that SRL in BL requires a more holistic account than purely cognitive models allow. Trautner et al. (2025), in their systematic review, note that although self-regulation is understood as context-dependent, the specific contextual features of digital and blended environments remain under-examined a gap our qualitative findings help to illustrate empirically, and which points to the need for theoretical models and research designs on SRL that integrate motivational, cognitive, emotional, and contextual dimensions.

#### 4.1.2 Strategies for SRL in BL contexts

The strategy repertoire identified in our focus groups shows that students were not passive recipients of the blended modality but deployed adaptive mechanisms to manage their learning. The integrated-calendar use and the informal estimation and adjustment of cognitive resources that students described are consistent with Panadero's (2017) account of how such practices allow learners to refine expectations and plan study approaches with greater precision.

The prominence of peer interaction and collaboration in our data is consistent with the model of Socially Shared Regulation and the social scaffolding framework proposed by Hadwin et al. (2018), who distinguish three interrelated forms: (a) SRL, where the student plans, monitors, and adjusts their own learning; (b) CoRL (Co-Regulated Learning), where a peer or tutor temporarily scaffolds another student’s use of self-regulatory strategies; and (c) SSRL (Socially Shared Regulation of Learning), where a group jointly regulates goals, strategies, and monitoring. The instrumental collaboration our participants described (exchanging materials, resources, and notices) together with their use of peer and grade-based benchmarks, is consistent with these co- and socially-shared regulation processes, and suggests that LMS platforms can function as environments conducive to reflection when they surface clear, relevant indicators. We note, without being able to test this directly in our data, that emerging work on AI-enabled real-time support (Jarvela et al., 2023) and on detecting triggers for shared regulation (Dang et al., 2024; Järvelä and Hadwin, 2024) points to a possible avenue for extending this kind of scaffolding.

#### 4.1.3 Managing motivation for SRL in BL

The distinction our participants drew between activating and maintaining motivation is consistent with the literature’s conceptualization of motivation as a dynamic process (Pintrich and De Groot, 1990; Schunk et al., 2014). As in prior work on self-regulation, students described small achievements, rewards, and self-praise as motivational stimuli tied to micro-goals, self-concept, and sense of competence (Schunk et al., 2014; Pintrich and De Groot, 1990).

Participants’ accounts suggest that dismissing negative thoughts functioned, in their own descriptions, as a coping resource that supported motivation and self-regulation rather than as maladaptive avoidance. This is broadly consistent with Alonso-Tapia et al.'s (2020) model, in which emotional regulation is treated as part of a learning-oriented self-regulation style that helps sustain task engagement; although their model associates certain avoidance behaviors with less favorable outcomes, our participants’ accounts suggest that neutralizing negative thoughts can function adaptively during the self-reflection stage — a pattern based on self-report that would benefit from further verification beyond interview data. These considerations point to a possible direction for technological platforms: more systematically integrating motivational resources (reminders, reinforcement messages, progress visualizations) alongside organizational and monitoring features.

### 4.2 Design of the SRL app

#### 4.2.1 Expert review results

The expert evaluation identified both strengths and gaps in the application’s preliminary design. The higher achievement ratings in the planning stage are consistent with systematic reviews indicating that planning is the most tractable entry point for technology-mediated SRL support, involving concrete, implementable skills such as organizing materials and scheduling tasks (Edisherashvili et al., 2022; Luo and Zhou, 2024). The effort-, complexity-, and time-estimation functions added to the app were informed by Knight et al.'s (2022) finding that students tend to overestimate their performance and that more precise metacognitive calibration is associated with improved self-regulation; we treat this as the design rationale for the feature rather than as evidence, from our own data, that the feature achieved this calibration effect.

The low ratings for the self-reflection stage reflect a difficulty also reported elsewhere in technology-mediated SRL programs: sustaining deep reflective engagement. Consistent with reports that students engage more readily with functions offering immediate, tangible feedback than with reflection and self-motivation tasks requiring more scaffolding (Hilliger et al., 2020a; Sonderlund et al., 2018), our experts identified an absence of robust self-judgment and self-motivation features — a pattern that echoes prior observations that technological resources tend to prioritize organization and monitoring over motivational and reflective dimensions (Bergdahl et al., 2024; Pinheiro et al., 2021; Trautner et al., 2025).

The expert evaluation also pointed to a need to strengthen real-time self-monitoring and peer collaboration. The resulting design changes (pop-up notifications, progress visualizations, and a dedicated help-request button) were informed by recommendations on using LA to support co-regulation and socially shared regulation (Dang et al., 2024), consistent with evidence that progress indicators and socioemotional interaction are associated with higher engagement and self-efficacy (Heikkinen et al., 2025); we did not measure engagement or self-efficacy directly, so this connection is offered as design rationale rather than as a tested outcome of our study. In summary, expert review indicated that the preliminary app had greater functional density in the planning stage, less in performance, and the least in self-reflection an imbalance also reflected in student ratings (Section 4.2.2) and treated here as a design challenge for subsequent versions.

#### 4.2.2 Perceived impact and technological acceptability of the preliminary design

The perceived-impact results reported here correspond to the same preliminary version of the application evaluated by the expert reviewers, and offer a complementary, student-facing perspective on the same design. As in the expert evaluation, students rated performance-stage functions particularly those facilitating collaboration and resource exchange more positively than self-reflection functions, which received the lowest scores on the instrument. This convergence between expert and student ratings is consistent with prior work suggesting that self-evaluation and self-motivation are the most difficult SRL dimensions to operationalize through technology and require more explicit support (Edisherashvili et al., 2022; Luo and Zhou, 2024; Trautner et al., 2025).

The moderate ratings for planning functionalities suggest, on one hand, that the preliminary version offered resources students found somewhat useful for anticipating effort, time, and task complexity; on the other, that these functions were not yet experienced as fully consolidated or effective.

Regarding perceived usefulness and ease of use, students reported moderate overall acceptance, with usefulness rated more highly than usability. Under the Technology Acceptance Model, this pattern is generally considered favorable for adoption, since it suggests users perceived practical value despite usability friction (Alyoussef, 2023; Granic, 2022). That usefulness received the highest mean score on the entire scale is consistent with the possibility that grounding the app’s design in students’ own reported needs supported its perceived relevance (Barz et al., 2024; Vladova et al., 2021), though we note this is an inference from the perceived-usefulness ratings rather than a directly tested design effect.

The convergence between expert feedback and student perceptions of the preliminary version helped clarify which aspects of the application required further attention, but the two sources informed different types of refinement. Functional changes to Regula2 were driven by the expert review and included: (i) a weekly calendar view; (ii) the ability to add personal events; (iii) pre/post estimation and comparison of cognitive load (complexity, effort, time); (iv) pop-up self-monitoring reminders; and (v) an explicit peer help-request function. Pilot 1 did not lead to the addition or removal of core functionalities; instead, its results informed usability-oriented refinements to the interface, including visual hierarchy, element positioning, screen organization, color use, and navigation, with the aim of making the existing functions more intuitive and user-friendly.

### 4.3 Final design

The final design resulted from an iterative co-construction process involving experts and students, intended to situate the app in the actual practices and difficulties reported by students in blended contexts rather than in a purely theoretical translation of SRL constructs. This participatory approach is consistent with recommendations that such strategies support the relevance, acceptability, and potential adoption of educational technology by incorporating both functional and usability expectations of end users (Dang et al., 2024; Ifenthaler and Yau, 2020; Vladova et al., 2021).

The final version’s cognitive load estimation feature, weekly activity view, study-strategy repository, pop-up reminders, and help-seeking button reflect an effort to address the expert recommendations and the student needs identified in the qualitative stage. This is consistent with prior arguments that effective educational technology needs to integrate cognitive, social, and motivational dimensions of learning (Edisherashvili et al., 2022; Paulsen and Lindsay, 2024). Between Pilot 1 and Pilot 2, these core functionalities were retained; the subsequent refinement focused on interface organization, visual presentation, and navigation rather than on adding new self-regulatory functions. Whether the final design achieved this integration in practice is addressed, at the level of perceived impact, in Section 4.4.

Despite these design changes, an imbalance persisted in functional coverage across SRL phases: planning and performance remained overrepresented in the number and variety of interfaces relative to self-reflection, which remained the least developed area despite the gamification and estimation-comparison features introduced to address it. This is consistent with prior observations that reflective and motivational components are the most difficult SRL dimensions to operationalize through technology (Pinheiro et al., 2021; Trautner et al., 2025).

### 4.4 Perceived impact and technological acceptability of the final design

Students’ ratings of the final version were descriptively higher than those of the preliminary version across all three SRL phases (see Results). Because the core functional architecture was retained between Pilot 1 and Pilot 2, this pattern should not be interpreted as evidence that new self-regulatory functions produced the higher ratings. Rather, it is consistent with, though it does not on its own establish, the possibility that the combination of functional refinements introduced following expert review and subsequent usability-oriented interface refinements facilitated students’ access to and interaction with the existing SRL supports. Given the absence of a control group and the substantial difference in sample size between pilots (*n* = 246 vs. *n* = 21), we treat this as a plausible descriptive interpretation rather than a demonstrated effect. This interpretation is broadly consistent with literature suggesting that educational applications providing concrete tools for goal-setting, scheduling, and time management have the potential to support SRL processes (Luo and Zhou, 2024).

Relatedly, our finding that students rated the cognitive-load estimation items favorably in Pilot 2 is consistent with Knight et al.'s (2022) argument that more precise metacognitive calibration is associated with improved self-regulation, and with broader claims that blended-learning tools integrating cognitive, motivational, and contextual dimensions can extend the scope of SRL programs (Paulsen and Lindsay, 2024; Trautner et al., 2025). We note that our data speak to students’ perceived experience of these features, not to a direct measure of calibration accuracy.

In the performance stage, students rated collaborative and peer-support features favorably, consistent with the view that social interaction is a key motivational factor for sustaining learning in virtual and blended environments (Boekaerts and Corno, 2005). This is in line with prior work suggesting that collaborative components in educational applications are associated with co-regulation and socially shared regulation processes, which in turn relate to motivation and self-regulatory strategy use (Dang et al., 2024; Hadwin et al., 2018; Heikkinen et al., 2025) though we did not measure these downstream processes directly.

At the same time, the comparatively lower (though improved) ratings on self-monitoring items suggest that real-time metacognitive reflection remains an area for further development in the app. This is consistent with prior characterizations of this component as both harder to support technologically and central to consolidating autonomous self-regulation, since it enables students to adjust study methods in response to task demands (Hilliger et al., 2020b; Luo and Zhou, 2024). Recent work on LA and automated feedback suggests that personalized notifications and progress visualizations are associated with greater metacognitive awareness and more informed decision-making during task performance (Chen, 2024; Paulsen and Lindsay, 2024), which may represent a useful direction for strengthening this component in future versions.

Taken together, students’ more favorable ratings of collaborative features relative to individual monitoring features suggest that digital SRL tools may need to balance social and metacognitive components to support their perceived usefulness across dimensions. Advancing features that combine immediate feedback with explicit support for personal monitoring appears, on this basis, to be a promising direction for the performance phase of technology-mediated SRL tools.

In the self-reflection stage, students reported particularly valuing the ability to contrast their initial expectations of complexity, effort, and time with their actual experience. We interpret this as a meaningful contribution to the metacognitive dimension of the SRL model, consistent with the broader literature on metacognitive calibration and self-reflection (Alonso-Tapia et al., 2020; Knight et al., 2022) while noting that this is a perceived-impact finding rather than a direct measure of improved calibration or self-reflective skill. Students also reported a comparatively smaller, though still positive, contribution of the app to evaluating their own learning process and identifying their level of learning achieved, which reinforces the importance of this phase while pointing to room for improvement in supporting more precise self-evaluation.

Finally, consistent with technology acceptance literature (TAM; Davis, 1989), our finding that perceived usefulness exceeded perceived ease of use in Pilot 2 aligns with reports that students may tolerate usability friction when they perceive clear pedagogical value, though a persistent ease-of-use gap can still limit long-term adoption (Alyoussef, 2023; Barz et al., 2024; Granic, 2022). In the context of this study, the descriptively higher ease-of-use rating in Pilot 2 is also consistent with the intended purpose of the usability-oriented interface refinements implemented after Pilot 1; however, the non-equivalent pilot samples do not allow this difference to be attributed causally to those refinements. This suggests that, alongside pedagogical relevance, continued attention to usability and interface design remains important for the app’s sustained integration into students’ academic routines.

These findings suggest several implications for the design of technology-mediated SRL interventions in higher education. First, our results point to a continued need for explicit scaffolding of self-reflection particularly self-judgment and self-motivation dimensions that remained underserved across both expert review and student ratings in this study and are reported as similarly underserved elsewhere. Second, the comparatively lower ratings for self-monitoring functions suggest that real-time metacognitive feedback mechanisms, potentially supported by learning analytics, may be a useful direction for future design. Finally, the more favorable ratings for collaborative features relative to individual monitoring suggest that platforms may benefit from balancing social and individual metacognitive components rather than emphasizing one at the expense of the other.

It is important to clarify the nature of this study’s theoretical contribution. Rather than testing or extending Zimmerman’s model empirically, this study used the model as a functional classification framework to organize qualitative findings and guide application design — a theory-informed design contribution rather than an empirical theory-testing one. The descriptive pilot data presented here speak to students’ perceived experience of a theory-informed tool, not to whether learning analytics alter the underlying mechanisms of self-regulated learning.

This study has several limitations that should be considered when interpreting the results. The application’s validation was conducted in a limited set of courses and Chilean universities, which restricts the immediate generalizability of the findings to other institutional or cultural contexts. Differential analyses considering individual-level variables, such as prior engagement, technology experience, or disciplinary background were not performed and could have influenced perceived impact.

A methodological limitation of the study was that, due to the application’s design, usage logs could only be extracted in aggregate for all participants. This prevented the calculation of metrics differentiated by pilot and confirmation of whether students actively interacted with each function before evaluating it. Consequently, future studies should carefully design software architectures that guarantee individual traceability and accurate segmentation by course from the initial stages of technological implementation.

Additionally, the study did not include a control group, which precludes attributing the observed differences in perceived impact between pilots to the application itself rather than to other concurrent factors (e.g., instructor effects, course maturation, or the substantially smaller Pilot 2 sample, *n* = 21 vs. *n* = 246). The design was also cross-sectional within each pilot, with no longitudinal follow-up to assess whether perceived impact was sustained over time. Consequently, the findings cannot support causal claims about the application’s effect on the development of self-regulated learning; they should be interpreted strictly as descriptive, perceived-impact evidence from two non-equivalent implementations, rather than as confirmatory evidence of the app’s effectiveness.

Based on these considerations, several directions for future research are identified. One is the development of self-reflection modules supported by guided prompts and formative rubrics offering concrete guidance for critical reflection on performance. The integration of artificial intelligence may offer a promising avenue for personalized adaptive support and for detecting triggers for co-regulation and shared regulation processes in real time. Another direction involves evaluating the application’s association with academic outcomes and student well-being indicators, considering both cognitive and socioemotional dimensions. Finally, we echo the need for controlled, quasi-experimental designs with larger and more comparable samples across pilots, which would allow for a more rigorous estimation of the application’s association with self-regulation and the educational experience in blended learning environments, and for a genuine test of the causal claims that the present descriptive design cannot support.

## 5 Conclusion

This study reported the design, iterative validation, and preliminary evaluation of Regula2, a student-facing learning analytics application integrated into an LMS virtual classroom, developed to support self-regulated learning across Zimmerman’s three phases in blended higher education. Three contributions emerge from the work.

The findings indicate that SRL in mixed modalities is shaped not only by individual skills but also by contextual factors, such as digital resource overload, limited timely feedback, and the need to sustain habits of self-care and motivation. At the same time, students deployed adaptive strategies, both individual and collaborative, in line with models of co-regulation and socially shared regulation, underscoring the importance of integrating cognitive, motivational, and social dimensions in the design of pedagogical supports.

Likewise, the integration of qualitative findings, expert review, and student ratings showed that the functional design of Regula2 provided clearer support for the planning and performance phases of SRL than for self-reflection. Features such as cognitive load estimation, a weekly calendar, task lists, and pop-up reminders were positively perceived, although these results reflect perceived support rather than measured improvement in students’ self-regulatory skills. Insufficient coverage of the self-reflection (self-evaluation) phase remained evident across expert review and student ratings, pointing to a persistent gap in supporting self-evaluation and self-motivation processes, both considered essential for closing the self-regulatory cycle.

Finally, technology acceptance was moderate in Pilot 1 and descriptively higher in Pilot 2, with perceived usefulness exceeding perceived ease of use in the final implementation. Because the core functional architecture was maintained between the two pilots, while Pilot 2 incorporated usability-oriented refinements to visual presentation, element positioning, screen organization, visual hierarchy, and navigation, the higher ratings should not be interpreted as the effect of adding new functionalities. Rather, they are consistent with the possibility that a more intuitive and user-friendly interface facilitated students’ access to and interaction with the existing functions. Given the non-equivalent samples and implementation contexts, this interpretation remains descriptive and does not support a causal attribution to the interface refinements. These findings point to the value of continuing to develop real-time metacognitive feedback mechanisms and a better balance between social and reflective components in future iterations.

Together, these findings support the case for student-facing learning analytics as a theoretically grounded and promising approach to supporting self-regulation in digitally mediated higher education, one that positions students as active agents in interpreting their own learning data rather than passive subjects of instructor-facing monitoring systems. Confirming this promise, however, will require larger and more comparable samples, controlled or quasi-experimental designs, and longitudinal evidence capable of examining whether perceived support translates into sustained changes in self-regulated learning.

## Data Availability

The original contributions presented in the study are included in the article/Supplementary material, further inquiries can be directed to the corresponding author/s.
