# Supplementary material for: Designing for self-regulation: development and preliminary user evaluation of a student-facing learning analytics app in blended higher education
Source: Front Psychol. 2026 Sep 14;17:1914516. doi: 10.3389/fpsyg.2026.1914516 (PMC13616683; doi:10.3389/fpsyg.2026.1914516)
Supplement: Supplementary file 1 [file Table_1.DOCX]

Supplementary Material

**Regula2: Designing for Self-Regulation in Blended Higher Education**

*This Supplementary Material contains item-level descriptive statistics for the Scale for Perceived Impact and Technological Acceptability of the SRL App, administered in Pilot 1 (preliminary version) and Pilot 2 (final version). Tables are organized by SRL phase (Forethought, Performance, Self-Reflection) and by Technology Acceptance Model (TAM) items.*

# 1 Supplementary Data

## Table 1. Pilot 1 Forethought phase item-level descriptive statistics

| **Item** | **M** | **SD** | **Min** | **Max** |
| --- | --- | --- | --- | --- |
| Organizing my time | 2.56 | 1.97 | 1 | 5 |
| Organizing my activities | 2.58 | 1.97 | 1 | 5 |
| Anticipating the study strategy | 2.65 | 2.09 | 1 | 5 |
| Estimating the complexity of academic activities | 2.72 | 2.19 | 1 | 5 |
| Estimating the amount of time required to complete an academic activity | 2.74 | 2.24 | 1 | 5 |
| Estimating the amount of effort required to complete the activity | 2.86 | 2.35 | 1 | 5 |
| Identifying study techniques to complete an academic activity | 2.77 | 2.30 | 1 | 5 |

*Note. M = Mean; SD = Standard Deviation; Min = Minimum; Max = Maximum.*

## Table 2. *Pilot 1 Performance phase item-level descriptive statistics*

| **Item** | **M** | **SD** | **Min** | **Max** |
| --- | --- | --- | --- | --- |
| Exchanging resources with my classmates (files, external pages, videos, among others) | 2.88 | 2.55 | 1 | 5 |
| Sharing resources or materials that I considered important for an academic activity | 2.98 | 2.49 | 1 | 5 |
| Learning about the study techniques used by my classmates | 2.91 | 2.53 | 1 | 5 |
| Asking classmates for help through the app | 2.63 | 2.29 | 1 | 5 |
| Monitoring the way in which I am learning | 2.77 | 2.38 | 1 | 5 |
| Identifying if the study strategies I am using are helping me to study | 2.77 | 2.38 | 1 | 5 |

*Note. M = Mean; SD = Standard Deviation; Min = Minimum; Max = Maximum.*

## Table 3. Pilot 1 Self-reflection phase item-level descriptive statistics

| **Item** | **M** | **SD** | **Min** | **Max** |
| --- | --- | --- | --- | --- |
| Evaluating my learning process after finishing an academic activity | 2.23 | 1.57 | 1 | 5 |
| Identifying my level of learning after finishing an academic activity | 2.30 | 1.63 | 1 | 5 |
| Comparing if the level of complexity I estimated before the activity was accurate | 2.35 | 1.72 | 1 | 5 |
| Comparing if the level of effort I estimated before the activity was accurate | 2.33 | 1.73 | 1 | 5 |
| Comparing if the dedicated time I estimated before starting the activity was accurate | 2.37 | 1.69 | 1 | 5 |

*Note. M = Mean; SD = Standard Deviation; Min = Minimum; Max = Maximum.*

## Table 4. Pilot 1 Technology Acceptance Model (TAM) items

| **Item** | **M** | **SD** | **Min** | **Max** |
| --- | --- | --- | --- | --- |
| I found the app easy to use | 2.86 | 2.41 | 1 | 5 |
| I found the app useful | 3.21 | 2.54 | 1 | 5 |

*Note. M = Mean; SD = Standard Deviation; Min = Minimum; Max = Maximum.*

## Table 5. Pilot 2 Forethought phase item-level descriptive statistics

| **Item** | **M** | **SD** | **Min** | **Max** |
| --- | --- | --- | --- | --- |
| Organizing my time | 4.5 | 0.7 | 1 | 5 |
| Organizing my activities | 4.8 | 0.5 | 1 | 5 |
| Anticipating the study strategy | 4.1 | 0.9 | 1 | 5 |
| Estimating the complexity of academic activities | 4.0 | 0.8 | 1 | 5 |
| Estimating the amount of time required to complete an academic activity | 4.1 | 0.9 | 1 | 5 |
| Estimating the amount of effort required to complete the activity | 4.0 | 0.9 | 1 | 5 |
| Identifying study techniques to complete an academic activity | 4.0 | 1.2 | 1 | 5 |

*Note. M = Mean; SD = Standard Deviation; Min = Minimum; Max = Maximum.*

## Table 6. Pilot 2 Performance phase item-level descriptive statistics

| **Item** | **M** | **SD** | **Min** | **Max** |
| --- | --- | --- | --- | --- |
| Exchanging resources with my classmates (files, external pages, videos, others) | 4.4 | 0.7 | 1 | 5 |
| Sharing resources or materials that I considered important for an academic activity | 4.4 | 0.7 | 1 | 5 |
| Learning about the study techniques used by my classmates | 3.7 | 1.1 | 1 | 5 |
| Asking classmates for help through the app | 4.2 | 0.7 | 1 | 5 |
| Monitoring the way in which I am learning | 3.9 | 1.1 | 1 | 5 |
| Identifying if the study strategies I am using are helping me to study | 3.8 | 1.2 | 1 | 5 |

*Note. M = Mean; SD = Standard Deviation; Min = Minimum; Max = Maximum.*

## Table 7. *Pilot 2 Self-reflection phase and Technology Acceptance Model (TAM) items*

| **Item** | **M** | **SD** | **Min** | **Max** |
| --- | --- | --- | --- | --- |
| Evaluating my learning process after finishing an academic activity | 3.8 | 1.3 | 1 | 5 |
| Identifying my level of learning after finishing an academic activity | 3.7 | 1.5 | 1 | 5 |
| Comparing if the level of complexity I estimated before the activity was accurate | 4.4 | 0.6 | 1 | 5 |
| Comparing if the level of effort I estimated before the activity was accurate | 4.4 | 0.7 | 1 | 5 |
| Comparing if the dedicated time I estimated before starting the activity was accurate | 4.4 | 0.6 | 1 | 5 |
| I found the app easy to use (TAM) | 3.8 | 1.2 | 1 | 5 |
| I found the app useful (TAM) | 4.1 | 1.2 | 1 | 5 |

*Note. M = Mean; SD = Standard Deviation; Min = Minimum; Max = Maximum. Pilot 1: n = 246 students, 5 courses. Pilot 2: n = 21 students, 1 course.*
